# Circ-ITCH sponges miR-214 to promote the osteogenic differentiation in osteoporosis via upregulating YAP1

**DOI:** 10.1038/s41419-021-03586-y

**Published:** 2021-04-01

**Authors:** Da Zhong, Gan-Zhe Xu, Ju-Zhi Wu, Hua Liu, Ju-Yu Tang, Cheng-Gong Wang

**Affiliations:** 1 Department of Orthopaedics, Xiangya Hospital of Central South University, 410008 Changsha, Hunan Province People’s Republic of China; 2 Hunan Key Laboratory of Aging Biology, Xiangya Hospital, Central South University, 410008 Changsha, Hunan Province People’s Republic of China; 3 Shanghai Medical College of Fudan University, 200000 Shanghai, People’s Republic of China; 4 Changsha Medical University, 410008 Changsha, Hunan Province People’s Republic of China

**Keywords:** Cell biology, Diseases

## Abstract

Osteoporosis is the most prevailing primary bone disease and a growing health care burden. The aim of this study was to clarify the functional roles and mechanisms of the circ-ITCH regulating osteogenic differentiation of osteoporosis. Circ-ITCH and yes-associated protein 1 (YAP1) levels were downregulated, but the miR‐214 level was upregulated in osteoporotic mice and patients. Knockdown of circ-ITCH inhibited the alkaline phosphatase (ALP) activity, mineralized nodule formation, and expression of runt-related transcription factor 2 (RUNX2), osteopontin (OPN), and osteocalcin (OCN) during osteogenic induction. Furthermore, miR-214 was a target of circ-ITCH, knockdown of miR-214 could impede the regulatory effects of sh-circ-ITCH on osteogenic differentiation. Moreover, miR-214 suppressed hBMSCs osteogenic differentiation by downregulating YAP1. Finally, in vivo experiments indicated that overexpression of circ-ITCH could improve osteogenesis in ovariectomized mice. In conclusion, circ-ITCH upregulated YAP1 expression to promote osteogenic differentiation in osteoporosis via sponging miR-214. Circ-ITCH could act as a novel therapeutic target for osteoporosis.

## Introduction

Osteoporosis is one of the most common bone diseases with bone loss and reduced bone mineral density (BMD), which increases bone fragility and occurrence of fracture^1^. In fact, osteoporotic fracture usually results in lasting disability and mortality in the elderly population and postmenopausal women^2^. In China, it is predicted that at least 90 million people suffer from osteoporosis^3^. Because of the high prevalence rate, there is a great demand to find effective methods of osteoporosis prevention and treatment. Studies have shown that in people with osteoporosis, bone loss is induced by the reduced bone formation and increased bone resorption^4,5^. The bone formation could be modulated by many transcription factors and signaling pathways^6–8^. Therefore, researching the mechanisms that modulate bone formation will be significant for the treatment of osteoporosis.

Among the non-coding RNAs, circular RNAs (circRNAs) are highly abundant and evolutionarily conserved endogenous RNAs with a covalently closed loop^9^. Recent evidences have revealed that circRNAs participate in cell growth, migration, invasion, and apoptosis^10–12^. Circ-ITCH is a well-known circRNA that spans several exons of itchy E3 ubiquitin-protein ligase (ITCH), it exerts inhibitory effects in many cancers through sponging certain miRNAs^13,14^. Recently, it was reported that during periodontal ligament stem cell (PDLSC) osteogenic differentiation, circ-ITCH was upregulated and might regulate the osteogenic differentiation through MAPK pathway^15^. However, to date, the effects of circ-ITCH in osteoporosis remain largely unclear and need further investigation. Accumulating evidences suggest the critical roles of miR-214 in bone formation^16,17^. For example, miR-214 negatively regulated osteogenic differentiation of hPDLSCs through inactivating transcription factor 4^17^. MiR-214 suppressed the osteogenic differentiation of bone marrow-derived mesenchymal stem cells (BMSCs) through inhibiting JNK and p38 pathways^18^. Furthermore, it was reported that miR-214 contained complementary sequences to circ-ITCH, and circ-ITCH served tumor-suppressive roles in gliomas by targeting miR-214^19^. In addition, our bioinformatic analysis (StarBase; http://starbase.sysu.edu.cn/index.php) showed there were binding sites between circ-ITCH and miR-214. However, it is still unknown whether miR-214 could be regulated by circ-ITCH to mediate osteoporosis progression. Thus, in this study, we hypothesize that circ-ITCH may be involved in osteoporosis and may regulate the hBMSCs osteogenic differentiation by interacting with miR-214.

Recent studies suggest that yes-associated protein 1 (YAP1), an important downstream effector of the Hippo signaling pathway, has crucial roles in cell growth, differentiation, and organ size control^20,21^. In particular, YAP1 has been implicated as a regulator of osteoblast differentiation. For example, upregulation of YAP1 could promote mesenchymal stem cells to differentiate into osteocytes^22^. In addition, YAP1 promoted osteogenesis by interacting with β-catenin in osteoblast-lineage cells^23^. Moreover, knocking down YAP1 in mice using *Dmp1-Cre* decreased osteoblast number and bone mass^24^. However, the function of YAP1 on the osteogenic differentiation of hBMSCs in osteoporosis remains further investigated. Interestingly, bioinformatics analysis (StarBase) found putative YAP1 response elements on miR-214. But whether miR-214 could associate with YAP1 has never been reported. Therefore, we focus on the interaction between miR-214 and YAP1 in hBMSCs osteogenic differentiation in osteoporosis.

In this study, we firstly found that circ-ITCH was down‐regulated in osteoporosis but up‐regulated in osteogenic differentiation of hBMSCs. We demonstrated that circ-ITCH sponged miR‐214 to indirectly up‐regulate YAP1, thus promoting osteogenic differentiation in vitro and in vivo. This research may provide a new target for regulating the osteogenic ability of hBMSCs and improve the therapeutic effect for osteoporosis.

## Materials and methods

### Clinical samples and isolation of hBMSCs

All procedures were approved by the Medical Ethics Committee of the Xiangya Hospital, Central South University (Changsha, Hunan, China). Bone marrow samples were obtained with informed consent from 15 female postmenopausal osteoporosis patients and 15 postmenopausal women without osteoporosis recruited from Xiangya Hospital, Central South University. Osteoporosis was diagnosed using the World Health Organization parameters. Primary hBMSCs were separated from bone marrow samples as previously described^25^.

### Cell culture

The cell line of human BMSCs and 293T cells were obtained from the American Type Culture Collection (ATCC, Manassas, VA, USA). All the cell lines included in this study have been authenticated by STR profiling and tested for mycoplasma contamination. Cells were cultured in Dulbecco’s modified Eagles medium (DMEM, Gibco, Grand Island, NY, USA) supplemented with 10% fetal bovine serum (FBS, Invitrogen, Carlsbad, CA, USA), and 1% penicillin-streptomycin (Invitrogen) at 37 °C in a humidified condition of 5% CO_2_.

### Osteogenic differentiation

Osteogenic differentiation assay of cultured hBMSCs was performed as described^26^. Briefly, hBMSCs were seeded in 24-well plates (5 × 10^4^ cells/well) and grown to 80% confluence. Then hBMSCs were treated with a medium containing 200 μM ascorbic acid, 10 mM β-glycerophosphate, and 100 nM dexamethasone (all from Sigma, St Louis, MO, USA) for 14 days to induce osteogenic differentiation, and induction medium was replaced every 3 days.

### Lentiviruses infection and cell transfection

sh-circ-ITCH, circ-ITCH overexpression (OE-circ-ITCH) and YAP1 overexpression (OE-YAP1) lentiviruses, miR-214 mimics, miR-214 inhibitor, and their negative controls (NC) were all obtained from GenePharma (Shanghai, China). To generate lentiviruses, 293T cells were co-transferred with pGLVH1/GFP/Puro (GenePharma) and the packaging plasmids Helper 1.0 (GeneChem, Shanghai, China). Then lentiviruses were harvested and purified 72 h after transfection. The hBMSCs were seeded in 24-well plates to reach about 40% confluence and then infected with lentiviruses (multiplicity of infection of 50) in the presence of polybrene (5 μg/mL, GeneChem). Stably transfected hBMSCs were selected by 1 μg/mL puromycin (Invitrogen). The sequences of miR-214 mimics, miR-214 inhibitor, and their NC were as follows:

MiR-214 mimics: sense, 5′-ACAGCAGGCACAGACAGGCAG-3′;

antisense, 5′-GCCUGUCUGUGCCUGCUGUUU-3′.

Mimics NC: sense, 5′-UUCUCCGAACGUGUCACGUTT-3′;

antisense, 5′-ACGUGACACGUUCGGAGAATT-3′.

MiR-214 inhibitor: 5′-ACUGCCUGUCUGUGCCUGCUGU-3′.

Inhibitor NC: 5′-CAGUACUUUUGUGUAGUACAA-3′.

### Luciferase reporter assay

The potential binding sites between circ-ITCH and miR-214, miR-214, and YAP1 were predicted through StarBase online website (http://starbase.sysu.edu.cn/index.php). The 3′-UTR fragment of circ-ITCH and YAP1 containing the wild-type or mutant miR-214 binding sites were synthesized and inserted into PsiCHECK-2 vectors (Promega, Madison, WI, US), named as circ-ITCH-WT, circ-ITCH-MUT, YAP1-WT, or YAP1-MUT. hBMSCs and 293T cells were cultured in 6-well plates to reach about 70% confluence. Then cells were co-transfected with circ-ITCH-WT, circ-ITCH-MUT, YAP1-WT, or YAP1-MUT vector and either miR-214 mimics or miR-214 inhibitor using Lipofectamine® 2000 (Invitrogen). The luciferase activity in each group was determined on a Dual-Luciferase Assay System (Promega) after transfection 48 h.

### RNA immunoprecipitation (RIP) assay

hBMSCs and 293T cells were transfected with miR-214 inhibitor or inhibitor NC for 24 h. RIP assay was conducted with an EZ-Magna RIPTM RNA-binding Protein Immunoprecipitation Kit (Millipore Corporation, USA). Antibodies against Ago2 (#ab186733, 1:50, Abcam, Cambridge, UK) and IgG (#ab205718, 1:100, Abcam) were used for RIP. Finally, the immunoprecipitated RNAs were extracted and quantified by RT-qPCR.

### Alizarin red S staining and quantification

After osteogenic differentiation, hBMSCs were fixed using 70% ethanol, incubated with 2% Alizarin red staining reagent (Sigma) at room temperature (RT) for 20 min. Subsequently, hBMSCs were washed using phosphate-buffered saline (PBS, Invitrogen), and visualized using a light microscope (Zeiss, Germany). The accessing process was conducted by an assessor blind to treatment allocation. For Alizarin Red S quantification, the stains were dissolved in 1 mL cetylpyridinium chloride buffer (Sigma) for 1 h and the absorbance was measured at 562 nm. The level of mineralization was calculated based on the Alizarin red S standard curve and represented as µmol/µg protein.

### Alkaline phosphatase (ALP) staining and quantification

After osteogenic differentiation, hBMSCs were fixed using 4% PFA and stained using an ALP staining Kit (#RE051, GeFan Biotechnology Technology, shanghai, China). After washing with PBS, stains were visualized with a microscope (Zeiss, Germany). The accessing process was conducted by an assessor blind to treatment allocation. For ALP activity assay, hBMSCs were lysed by 1% Triton X-100 (Sigma) for 15 min, the supernatant was collected through centrifugation (14,000 × *g*, 5 min), ALP activity was measured by an ALP Colorimetric Assay Kit (BioVision, Milpitas, CA, USA) on a microplate reader at 405 nm.

### Ovariectomy (OVX) animal model

Sixty healthy 8-week-old C57BL/J6 female mice were obtained from SLAC Laboratory Animal Company, Ltd. (Shanghai, China) and housed in standard pathogen-free conditions. Mice were randomly divided into the following groups: Sham, OVX, OVX + OE-NC, and OVX + OE-circ-ITCH groups. After 1 week of acclimatization, mice were anesthetized with 5% ketamine and underwent bilateral OVX or sham operation in a bioclean environment as previously reported^27^. One week after OVX surgery, circ-ITCH overexpression lentiviral vector (OE-circ-ITCH) or negative control vector (OE-NC) was injected into OVX mice through tail intravenous injection once per week for 12 weeks. After 13 weeks of OVX surgery, the femurs of mice were harvested to measure the degree level of osteoporosis. BMD was measured using Dual-energy X-ray absorptiometry (Hologic, Bedford, MA, USA). The accessing process was conducted by an assessor blind to treatment allocation. All procedures were approved by the Institutional Animal Care and Use Committee of the Xiangya Hospital, Central South University (Changsha, Hunan, China).

### Hematoxylin and eosin (H&E) staining and Alcian blue staining

Femur samples from mice were fixed in 4% PFA overnight and decalcified in 10% ethylenediamine tetra-acetic acid for 30 days. Then paraffin-embedded samples were sectioned into 5-μm-thick slices. Then slices were treated with gradient ethanol and stained by H&E (Nanjing Jiancheng Bioengineering Institute, Nanjing, Jiangsu, China) or 1% Alcian Blue (Abcam) at RT. Images from each group were photographed with a microscope (Zeiss, Germany). The accessing process was conducted by an assessor blind to treatment allocation.

### Reverse transcription-quantitative polymerase chain reaction (RT-qPCR)

Total RNA was isolated from bone marrow samples and hBMSCs with TRIzol Reagent (Invitrogen). Then first-strand cDNA synthesis was produced using the PrimeScript RT reagent Kit (for mRNAs, Takara, Dalian, China) or TaqMan MicroRNA Reverse Transcription Kit (for miR-214, ThermoFisher Scientific). The qPCR was conducted with the SYBR Premix EX Taq Kit (Takara) on an ABI 7500HT real‑time PCR system. Glyceraldehyde-3-phosphate dehydrogenase (GAPDH) or U6 small nuclear RNA (U6 snRNA) was used as a control for mRNA or miRNA, respectively. The expression levels were measured through the 2^−ΔΔCt^ method. Primer sequences were synthesized by Sangon Biotech (Shanghai, China) as follows:

Circ-ITCH F: 5′-GCAGAGGCCAACACTGGAA-3′;

Circ-ITCH R: 5′-TCCTTGAAGCTGACTACGCTGAG-3′;

MiR-214 F: 5′-TGCCTGTCTACACTTGCTGTGC-3′,

MiR-214 R: 5′-GCGAGCACAGAATTAATACGAC-3′;

YAP1 F: 5′-TTCGGCAGGCAATACGGAAT-3′,

YAP1 R: 5′-GTTGAGGAAGTCGTCTGGGG-3′;

Osteocalcin (OCN) F: 5′-GGCAGCGAGGTAGTGAAGAG-3′,

OCN R: 5′-CTAGACCGGGCCGTAGAAG-3′;

Osteopontin (OPN) F: 5′-GATGGCCGAGGTGATAGTGT-3′,

OPN R: 5′-GTGGGTTTCAGCACTCTGGT-3′;

RUNX2 F: 5′-CGGAATGCCTCTGCTGTTAT-3′,

RUNX2 R: 5′-TTCCCGAGGTCCATCTACTG-3′;

GAPDH R: 5′-CCAGGTGGTCTCCTCTGA-3′,

GADPH F: 5′-GCTGTAGCCAAATCGTTGT-3′;

U6 R: 5′-CTCGCTTCGGCAGCACA-3′;

U6 F: 5′-AACGCTTCACGAATTTGCGT-3′.

### Western blot assay

Total protein was isolated from hBMSCs with RIPA lysis buffer (Beyotime, Shanghai, China) containing proteinase inhibitors (Sigma). Protein concentration was detected with an Enhanced BCA Protein Assay Kit (Beyotime). 20 µg total proteins were electrophoresed with 10% SDS-PAGE and then transferred onto PVDF membranes (Millipore, Burlington, MA, USA). After blocking by 5% non-fat milk for 2 h at RT, membranes were incubated with respective primary antibodies against YAP1 (13584-1-AP, 1:2000, Proteintech), RUNX2 (ab76956, 1:1000, Abcam), OPN (ab8448, 1:1500, Abcam), OCN (ab93876, 1:1000, Abcam) and GAPDH (ab181602, 1:5000, Abcam) at 4 °C overnight. Finally, membranes were incubated with the appropriate secondary HRP antibodies (ab7090 or ab190475, 1:5000, Abcam) for 2 h at RT. The membranes were exposed with chemiluminescence substance (ThermoFisher Scientific) and band intensity was measured by ImageJ software.

### Statistical analysis

All experiments have been conducted independently at least three times. All the data meet the assumption of normal distribution. Data were analyzed with Prism 6.0 (GraphPad Software, USA) and expressed as mean ± standard deviation (SD). Statistical analysis between two groups was performed using the Student’s *t*-test and multiple comparisons were carried out using one-way analysis of variance (ANOVA) followed by Tukey’s post hoc test. *P* < 0.05 was considered statistically significant.

## Results

### Circ-ITCH and YAP1 expressions were upregulated and miR-214 expression was downregulated during osteogenic differentiation

**Fig. 1:**
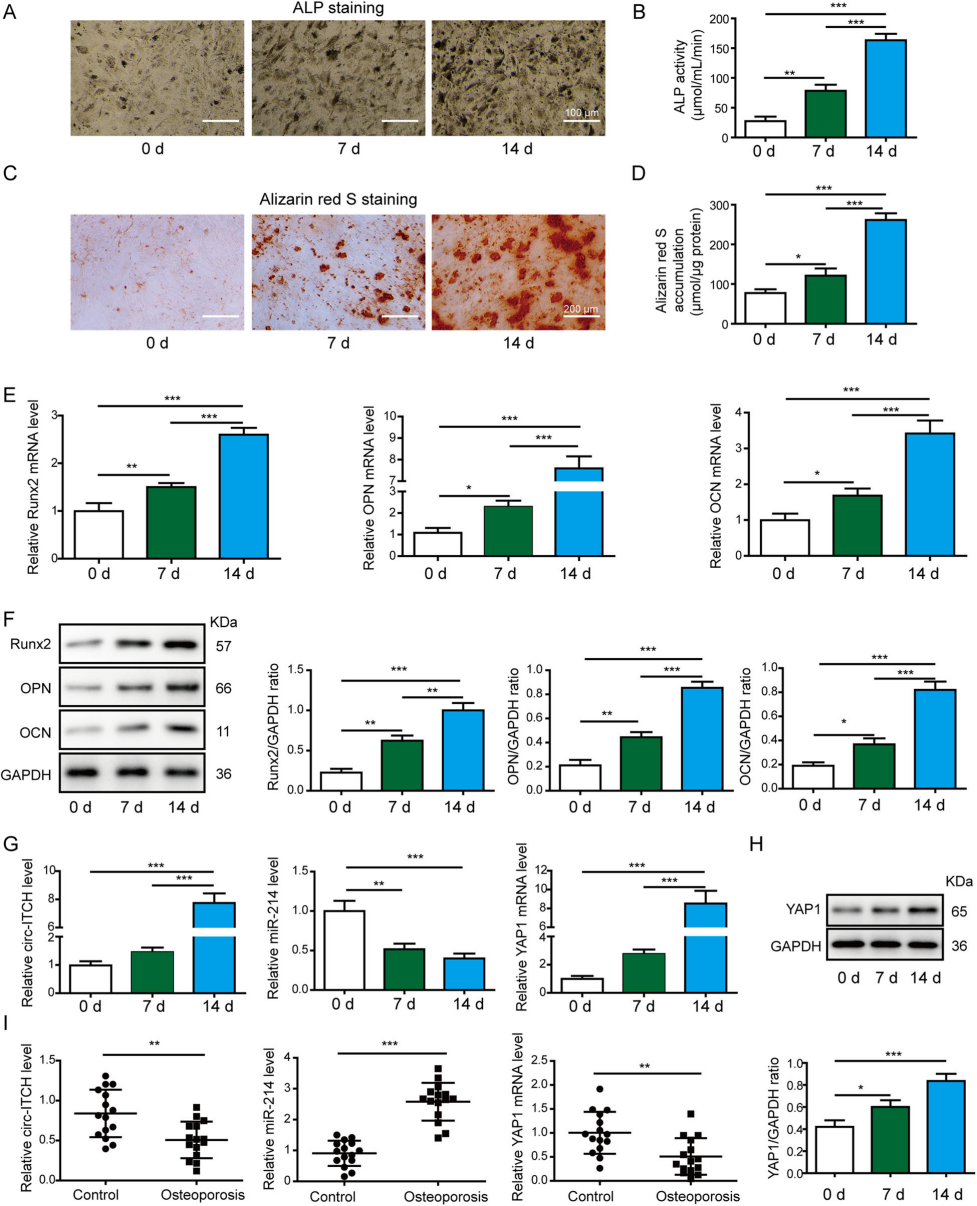
Circ-ITCH and YAP1 expressions were upregulated and miR-214 expression was downregulated during hBMSCs osteogenic differentiation. Osteogenic differentiation of hBMSCs was evaluated by ALP staining (**A** Scale bar: 100 μm) and ALP activity assay (**B**) at 0, 7, and 14 days after osteogenic induction. **C**, **D** Calcium deposition determined by Alizarin red S staining (Scale bar: 200 μm) and accumulation of mineralization on days 0, 7, and 14 after osteogenic induction. The mRNA and protein levels of osteogenic genes RUNX2, OPN, and OCN were determined by RT-qPCR (**E**) and western blot assay (**F**) at 0, 7, and 14 days. **G** The expression levels of circ-ITCH, miR-214, and YAP1 were determined by RT-qPCR after 0, 7, and 14 days of osteogenic culture. **H** The protein levels of YAP1 were determined by western blot assay after 0, 7, and 14 days of osteogenic culture. **I** Relative Circ-ITCH, miR-214, and YAP1 levels in 15 control (non-osteoporotic) samples and 15 osteoporotic samples. *P < 0.05, **P < 0.01, and ***P < 0.001.

After induction for 0, 7, and 14 days, the osteogenic capabilities of hBMSCs were evaluated by ALP staining and ALP activity assay for osteoblast differentiation, Alizarin red S staining, and quantification for calcium mineralization. We observed increased ALP staining (Fig. 1A, scale bar: 100 μm) and elevated ALP activity (Fig. 1B), as well as significantly higher levels of mineralized nodule formation (Fig. 1C, D, scale bar: 200 μm) at day 7 and 14, suggesting successful induction of osteogenic differentiation. Furthermore, we measured the expression of RUNX2, OPN, and OCN in hBMSCs. The mRNA (Fig. 1E) and protein (Fig. 1F) levels of these osteogenic markers were increased consistently throughout the differentiation process. Moreover, circ-ITCH expression level and YAP1 mRNA and protein levels were gradually increased, but the miR-214 expression was gradually reduced in a time-dependent manner during the osteogenic differentiation (Fig. 1G, H). Additionally, we measured the levels of circ-ITCH, miR-214, and YAP1 in control samples and osteoporotic samples. Circ-ITCH and YAP1 expressions were downregulated whereas miR‐214 expression was higher in osteoporotic samples (Fig. 1I). These data hinted that circ-ITCH, miR-214, and YAP1 may be involved in osteogenic differentiation of osteoporosis.

### Circ-ITCH promoted the osteogenic differentiation of hBMSCs in vitro

**Fig. 2:**
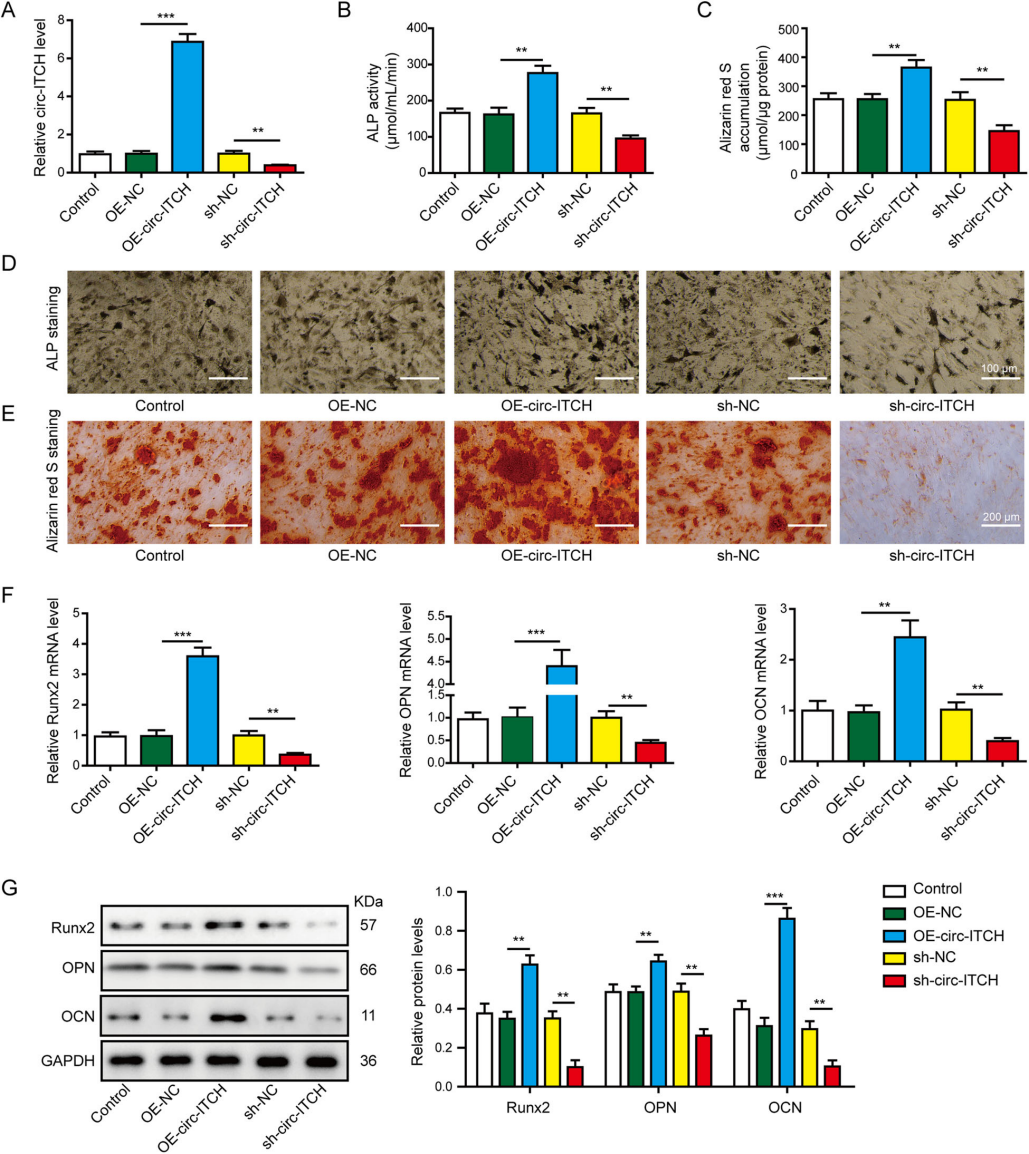
Circ-ITCH promoted the osteogenic differentiation of hBMSCs in vitro. hBMSCs were transfected by lentiviruses to overexpress circ-ITCH or knockdown circ-ITCH. **A** The transfection efficacy of OE-circ-ITCH and sh-circ-ITCH lentivirus vectors were verified by RT-qPCR. **B** ALP activity of hBMSCs at day 14 after osteogenic differentiation. **C** Alizarin red S accumulation in hBMSCs at day 14 after osteogenic differentiation. **D** ALP staining of hBMSCs at day 14 after osteogenic differentiation. Scale bar: 100 μm. **E** Alizarin red S staining in hBMSCs at day 14 after osteogenic differentiation. Scale bar: 200 μm. **F**, **G** The mRNA and protein levels of RUNX2, OPN, and OCN were determined by RT-qPCR and western blot assay at 14 day after osteogenic differentiation. **P < 0.01 and ***P < 0.001.

To examine the roles of circ-ITCH in osteogenic differentiation, lentiviruses infection in hBMSCs was performed to overexpress or knockdown its expression, respectively. The transfection efficiency was determined through RT-qPCR, circ-ITCH expression was markedly increased with transfection of OE-circ-ITCH and decreased with transfection of sh-circ-ITCH compared to their negative controls (Fig. 2A). The ALP activity (Fig. 2B) and ALP staining (Fig. 2D, scale bar: 100 μm), and mineralized nodule formation (Fig. 2C, E, scale bar: 200 μm) were significantly elevated by overexpression of circ-ITCH, whereas suppressed by knockdown of circ-ITCH after osteogenic differentiation for 14 days. Besides, the mRNA (Fig. 2F) and protein (Fig. 2G) levels of osteogenic markers RUNX2, OPN, and OCN were all induced by upregulating circ-ITCH, whereas were decreased by knocking down circ-ITCH. These results confirmed that circ-ITCH promoted the osteogenic differentiation of hBMSCs in vitro.

### Circ-ITCH directly targeted and suppressed miR-214 expression

**Fig. 3:**
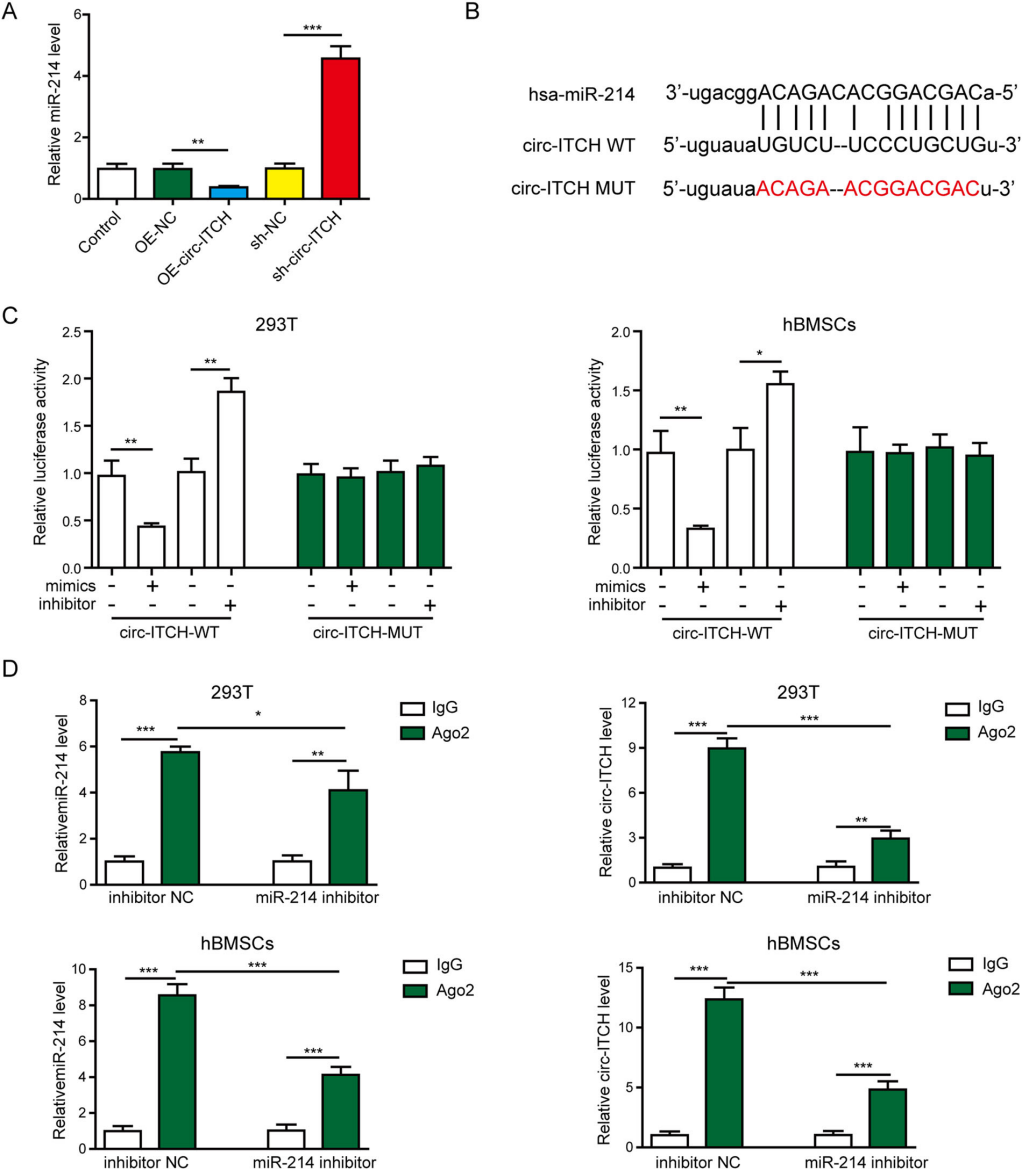
Circ-ITCH directly targeted and suppressed miR-214 expression. **A** hBMSCs were transfected with circ-ITCH overexpressing lentivirus, sh- circ-ITCH lentivirus or their negative controls for 48 h, and then expression level of miR-214 was measured by RT-qPCR. **B** StarBase analysis showed the potential binding sites for circ-ITCH and miR-214. **C** The luciferase activity was measured by dual-luciferase reporter assay in 293T cells and hBMSCs transfected with miR-214 mimics, miR-214 inhibitor or their negative controls for 48 h. **D** RIP assay was used to verify the association of circ-ITCH with miR-214 in 293T cells and hBMSCs. *P < 0.05, **P < 0.01, and ***P < 0.001.

The underlying mechanisms of circ-ITCH-mediated osteogenic differentiation were then explored. Overexpressing circ-ITCH decreased miR-214 while knocking down it increased miR-214 expression in hBMSCs (Fig. 3A). In addition, bioinformatics analysis (StarBase; http://starbase.sysu.edu.cn/index.php) by StarBase online website showed that the complementary sites between circ-ITCH and miR-214 (Fig. 3B). Then the targeted relationship between circ-ITCH and miR-214 was validated through a dual-luciferase reporter and RIP assays. The luciferase activity of circ-ITCH-WT was significantly lower when transfected with miR-214 mimics, but higher when transfected with miR-214 inhibitor (Fig. 3C). But, no significant changes were found in circ-ITCH-MUT groups (Fig. 3C). Also, circ-ITCH was specifically enriched in the Ago2 immunoprecipitates compared with control IgG (Fig. 3D). Moreover, miR-214 knockdown decreased the enrichment of circ-ITCH to Ago2 (Fig. 3D). These data revealed that circ-ITCH directly targeted and suppressed miR-214 expression.

### Circ-ITCH induced the osteogenic differentiation through directly targeting miR-214

**Fig. 4:**
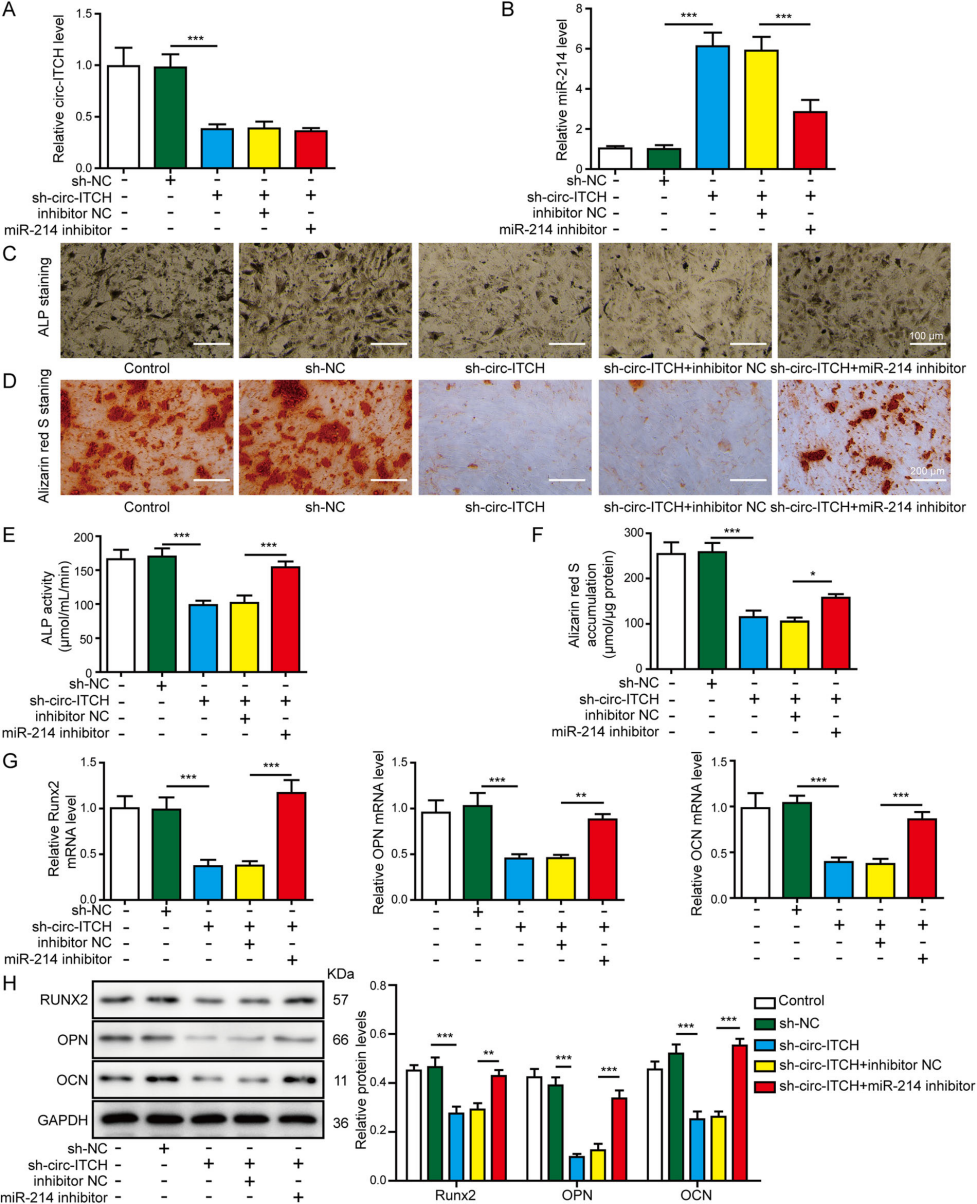
MiR-214 knockdown reversed the effects of circ-ITCH inhibition on osteogenic differentiation of hBMSCs. The hBMSCs were transfected with sh-circ-IITCH alone or co-transfected with sh-circ-IITCH and miR-214 inhibitor. **A**, **B** RT-qPCR analyses of circ-ITCH and miR-214 expression in hBMSCs. **C** ALP staining was performed on day 14 after osteogenic differentiation. Scale bar: 100 μm. **D** Alizarin red S staining was performed on day 14 after osteogenic differentiation. Scale bar: 200 μm. **E** ALP activity detection was performed on day 14 after osteogenic differentiation. **F** Alizarin red S accumulation detection was performed on day 14 after osteogenic differentiation. **G**, **H** The mRNA and protein expression levels of RUNX2, OPN, and OCN were assessed by RT-qPCR and western blot assays on day 14 after osteogenic differentiation. *P < 0.05, **P < 0.01, and ***P < 0.001.

We further explored whether circ-ITCH regulated the osteogenic differentiation via targeting miR-214. The hBMSCs were transfected with sh-circ-ITCH vector alone or co-transfected with miR-214 inhibitor, then circ-ITCH and miR-214 expressions were measured. As shown in Fig. 4A, B, sh-circ-ITCH transfection downregulated circ-ITCH expression and upregulated miR-214 expression, whereas co-silencing miR-214 downregulated miR-214 expression and had no effect on circ-ITCH expression. Functional studies showed that knockdown of miR-214 in hBMSCs could reverse the effects of sh-circ-ITCH on ALP staining (Fig. 4C, scale bar: 100 μm), ALP activity (Fig. 4E), and mineralized nodule formation (Fig. 4D, F, scale bar: 200 μm). Furthermore, the mRNA (Fig. 4G) and protein (Fig. 4H) levels of osteogenic markers RUNX2, OPN, and OCN were also reinforced by miR-214 inhibitor co-transfection compared to sh-circ-ITCH transfection alone. Taken together, circ-ITCH positively regulated the osteogenic differentiation of hBMSCs via directly targeting miR-214.

### MiR-214 directly and negatively regulated YAP1 expression

**Fig. 5:**
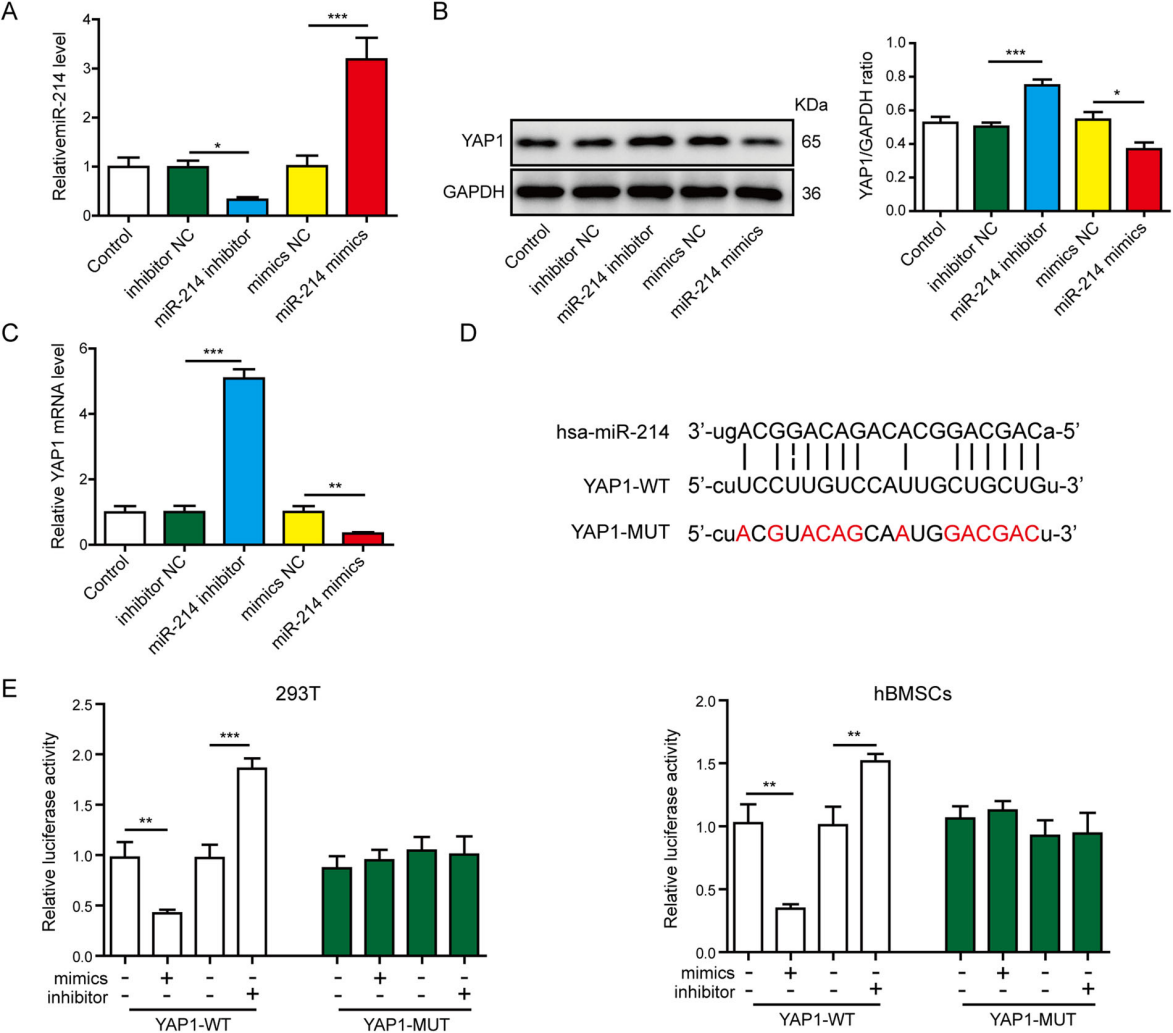
MiR-214 directly and negatively regulated YAP1 expression. The hBMSCs were transfected with miR-214 mimics, miR-214 inhibitor, or their negative controls for 48 h, then the expressions of miR-214 (**A**) and YAP1 (**B**, **C**) were measured by RT-qPCR and western blot. **D** StarBase analysis showed the potential binding sites for miR-214 and YAP1. **E** Dual-luciferase reporter assay was used to verify the association of miR-214 with YAP1 in 293T cells and hBMSCs. *P < 0.05, **P < 0.01, and ***P < 0.001.

Previous results indicated that YAP1 was an important modulator of osteogenic differentiation^28^. Next, we investigated whether YAP1 could be regulated by miR-214. After transfection with miR-214 mimics or inhibitors, the expression levels of miR-214 and YAP1 in hBMSCs were further detected. It was shown that the level of miR-214 was higher in miR-214 mimics group and lower in the miR-214 inhibitor group (Fig. 5A). The mRNA and protein levels of YAP1 were dramatically attenuated in the miR-214 mimics group, whereas their expression levels were obviously increased in the miR-214 inhibitor group (Fig. 5B, C). Then, the putative binding sites between miR-214 and YAP1 were predicted using StarBase (Fig. 5D). To further corroborate the specific interaction between miR-214 and YAP1, a dual-luciferase reporter assay was conducted. The luciferase activity in the YAP1-WT group was reduced by miR-214 mimics and increased by the miR-214 inhibitor, but no differences were revealed in the YAP1-MUT groups (Fig. 5E). Taken together, these data suggested that miR-214 was a negative regulator of YAP1.

### MiR-214 inhibited osteogenic differentiation through downregulating YAP1

**Fig. 6:**
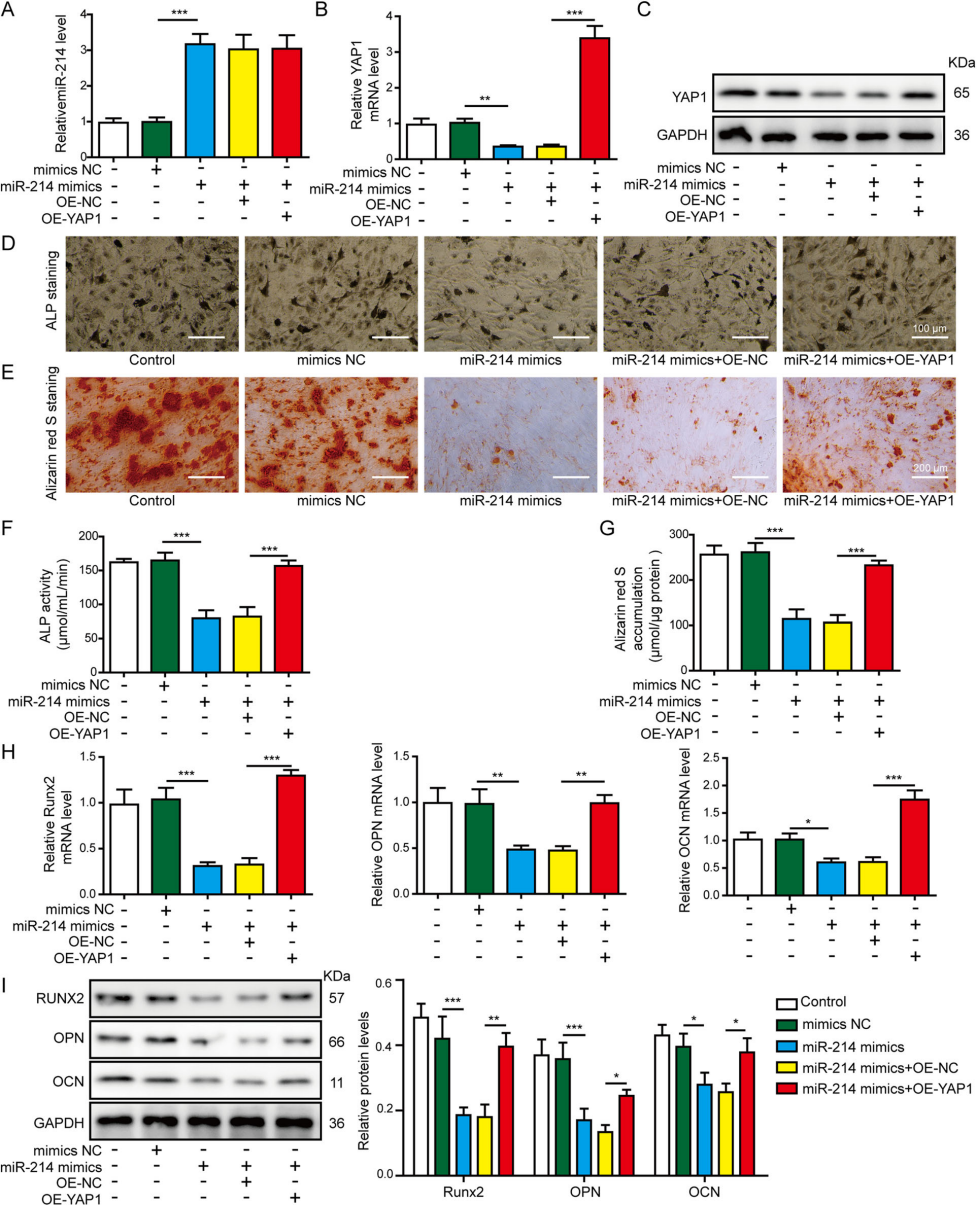
YAP1 reversed the effects of miR-214 overexpression on osteogenic differentiation of hBMSCs. The hBMSCs were transfected with miR-214 mimics alone or co-transfected with miR-214 mimics and OE-YAP1. **A**, **B** RT-qPCR analyses of miR-214 and YAP1 expression in hBMSCs. **C** The protein expression level of YAP1 in hBMSCs was assessed by western blot assay. **D** ALP staining detection was performed on day 14 after osteogenic differentiation. Scale bar: 100 μm. **E** Alizarin red S staining detection was performed to indicate calcification on day 14 of osteogenic differentiation. Scale bar: 200 μm. **F** ALP activity detection was performed on day 14 after osteogenic differentiation. **G** Alizarin red S quantification was performed to indicate calcification on day 14 of osteogenic differentiation. **H**, **I** The mRNA and protein expression levels of RUNX2, OPN, and OCN was assessed by RT-qPCR and western blot assays on day 14 after osteogenic differentiation. *P < 0.05, **P < 0.01, and ***P < 0.001.

To verify the roles of miR-214 on osteogenic differentiation and involvement of YAP1, hBMSCs were co-transfected with miR-214 mimics and the OE-YAP1 vector. As shown in Fig. 6A, C, miR-214 mimics downregulated the mRNA and protein levels of YAP1, while co-transfection of the OE-YAP1 vector upregulated the expression of YAP1, but had no effect on the miR-214 level. The ALP staining (Fig. 6D, scale bar: 100 μm), ALP activity (Fig. 6F), and mineralized nodule formation (Fig. 6E, G, scale bar: 200 μm) in hBMSCs were significantly decreased by overexpression of miR-214. However, YAP1 overexpression rescued the above effects induced by miR-214 mimics. Moreover, miR-214 overexpression also inhibited RUNX2, OPN, and OCN mRNA (Fig. 6H) and protein (Fig. 6I) levels. Furthermore, inhibited osteogenic markers mediated by miR-214 were significantly attenuated by OE-YAP1, as indicated by the upregulation of RUNX2, OPN, and OCN expression at both mRNA and protein levels (Fig. 6H, I). These results implied that miR-214 negatively regulated hBMSCs osteogenic differentiation by functionally targeting and inhibiting YAP1.

### Circ-ITCH overexpression alleviated the symptoms of osteoporosis in vivo

**Fig. 7:**
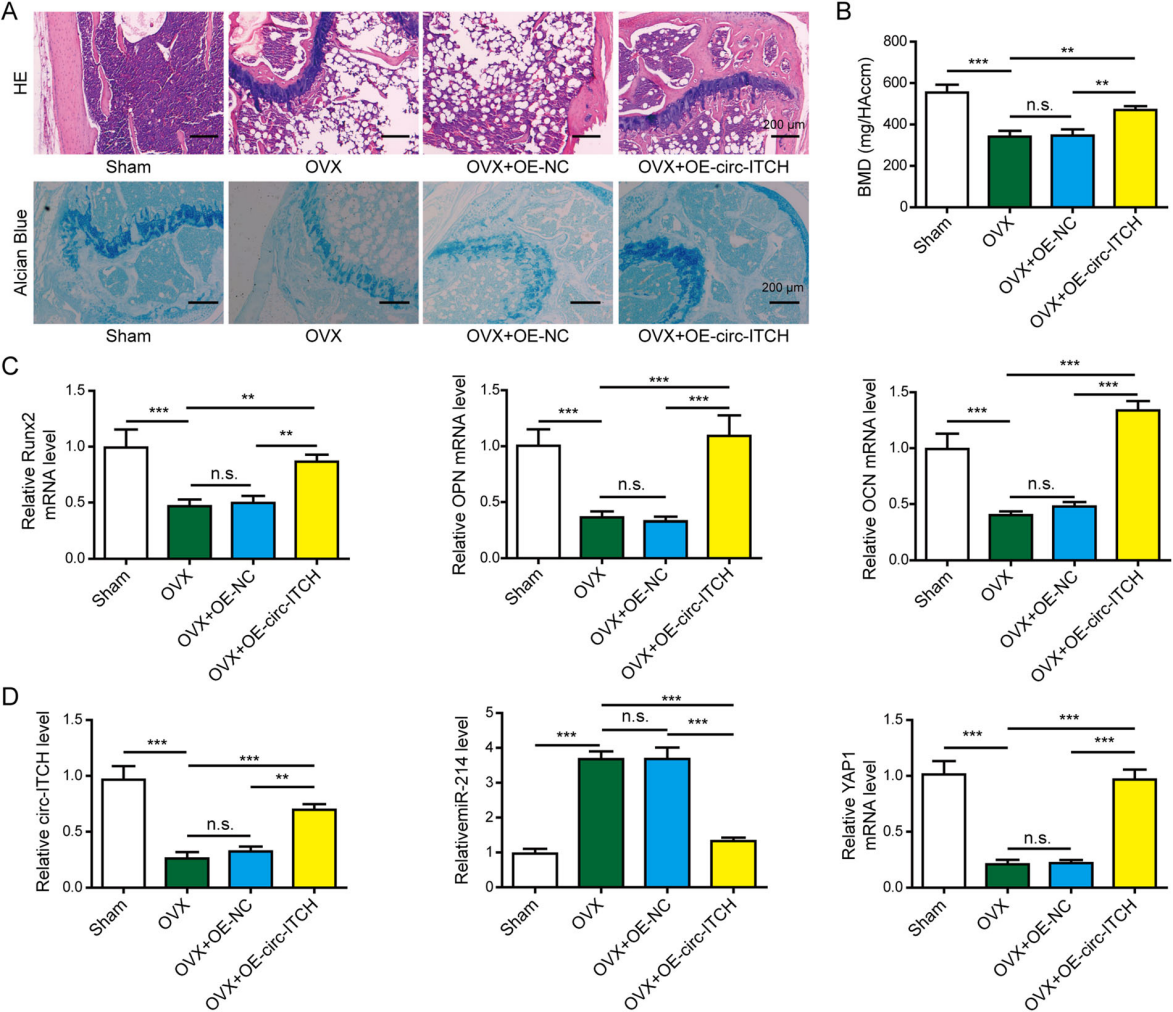
Overexpression of circ-ITCH alleviated the symptoms of osteoporosis in OVX mice. Mice were randomly divided into four groups: Sham, OVX, OVX + OE-NC, and OVX + OE-circ-ITCH groups. OE-circ-ITCH or OE-NC lentiviral vector was injected into OVX mice through tail intravenous injection. **A** Representative images of bone tissue sections after HE staining and Alcian Blue staining. Scale bar: 200 μm. **B** BMD of femurs was measured using Dual-energy X-ray absorptiometry. **C** RT-qPCR analysis of RUNX2, OPN, and OCN levels in the bone marrow samples. **D** RT-qPCR analysis of circ-ITCH, miR-214, and YAP1 levels in the bone marrow samples. **P < 0.01, ***P < 0.001, and n.s. not significant.

To determine whether circ-ITCH has the capability to ameliorate osteoporosis in vivo, we injected OE-ITCH or OE-NC lentiviral vector into OVX mice. Compared with the sham group, the OVX group showed the damaged bone tissue structures (Fig. 7A, scale bar: 200 μm) and apparent bone loss with decreased BMD (Fig. 7B), which indicated the model of the osteoporotic mice was successfully established. OVX-associated changes in the histological morphology were restored by circ-ITCH overexpression, showing the improved bone tissue structures (Fig. 7A, scale bar: 200 μm) and the increased BMD (Fig. 7B). The mRNA levels of RUNX2, OPN, and OCN were lower in the OVX group, while were upregulated by circ-ITCH overexpression compared with OVX and OVX + OE-NC groups (Fig. 7C). Moreover, OVX mice showed the decreased circ-ITCH and YAP1 expression levels and increased miR-214 expression (Fig. 7D). However, OVX mice injected with OE-circ-ITCH lentiviral vector indicated the increased expression of circ-ITCH and YAP1 while decreased miR-214 expression compared with OVX and OVX + OE-NC mice (Fig. 7D). Taken together, these results demonstrated circ-ITCH overexpression could prevent osteoporosis progression in vivo.

## Discussion

Osteoporosis is still a major medical challenge worldwide. In recent years, tissue regeneration through the differentiation of MSCs has been a prospective therapeutic approach to treat osteoporosis^29^. hBMSCs are typical adult MSCs with self‐renew capabilities and can differentiate into osteoblasts, which may be a potential cell source for bone tissue engineering^30^. The osteogenic differentiation of hBMSCs is vital for the application for bone regeneration^31^. Therefore, it is important to study the molecular mechanisms that control hBMSCs osteogenic differentiation. We demonstrated that overexpression of circ-ITCH effectively promoted hBMSCs osteogenic differentiation and prevented osteoporosis in the OVX mice model by targeting the miR-214/YAP1 axis. Our results provided new insight into the expression, function, and mechanism of circ-ITCH in osteogenic differentiation of osteoporosis.

Non-coding RNAs are important post-transcriptional regulators associated with the regulation of bone formation^32,33^. Previous studies have identified many circRNAs as biomarkers for osteoporosis, such as circ-RUNX2, circ-19142, and circ-5846^34–36^. For example, circRNA-28313 promoted OVX-induced bone resorption in mice by modulating miR-195a/CSF1 axis^33^. However, circRNA-0074834 could promote BMSCs osteogenic differentiation through sponging miR-942-5p^34^. Recently, many studies have verified that circ-ITCH has essential roles in the tumorigenesis and progression of human cancers^37^. For example, circ-ITCH reduced SP-1 expression via the PTEN/PI3K/AKT pathway, which suppressed proliferation, migration, and invasion of osteosarcoma cells^38^. Moreover, circ-ITCH might interact with miR-34a and miR-146a to regulate PDLSC osteogenic differentiation via the MAPK pathway^15^. These studies suggested that circ-ITCH might have clinical significance in bone regeneration and bone-related diseases. However, their regulatory functions and mechanisms need to be further investigated. In this study, we aimed at investigating the expression and function of circ-ITCH in osteoporosis. We observed that circ-ITCH was downregulated in bone marrow samples isolated from osteoporosis patients and OVX mice. Furthermore, circ-ITCH overexpression stimulated hBMSCs osteogenic differentiation in vitro and partially attenuated the bone loss of femurs and increased BMD values of OVX mice, suggesting that upregulation of circ-ITCH could prevent osteoporosis. Our results highlighted the potential of circ-ITCH in osteogenic differentiation and could potentially protect patients from osteoporosis. This is the first study reporting the expression level and role of circ-ITCH in osteoporosis.

Accumulating evidence suggested that miR-214 was implicated in osteogenic differentiation^39,40^. For example, miR-214 inhibited osteogenic differentiation of human PDLSCs by targeting ATF4 and suppressed BMSCs osteogenic differentiation through suppressing the JNK and p38 pathways^17,18^. Consistent with previous reports, we found that miR-214 level was higher in osteoporosis samples and overexpression of miR-214 inhibited hBMSCs osteogenic differentiation. We further researched the circ-ITCH-related molecular mechanisms during the regulation of hBMSCs osteogenic differentiation. Recently, circRNAs are recognized as miRNA sponges, reducing miRNA binding to its targets, to regulate biological processes. For example, circRNA-101368 regulated the migration of hepatocellular carcinoma via sponging miR-200a^41^. CircRNA-CBL.11 inhibited cell proliferation through targeting miR-6778-5p in colorectal cancer^42^. Moreover, circRNA-CDR1as promoted osteoblastic differentiation of periodontal ligament stem cells through the miR-7/GDF5/SMAD axis^19^. A recent study indicated that circ-ITCH suppressed glioma cancer through sponging miR-214^19^. Consistent with previous reports, we identified that circ-ITCH showed complementary sequences to miR-214, and validated the interaction between circ-ITCH and miR-214 using luciferase reporter and RIP assays. We also demonstrated that the effects of sh-circ-ITCH on hBMSCs osteogenic differentiation were attenuated by miR-214 inhibition. This is the first research reporting circ-ITCH could sponge miR-214 to promote osteogenic differentiation in osteoporosis.

MiRNAs have been reported to regulate numerous biological processes through binding to target mRNAs and silencing the gene expression^43^. YAP1, as a major downstream effector of the Hippo pathway, had a vital role in the adipo-osteogenic differentiation of human MSCs^44^. It could also promote osteogenic differentiation of MSCs and osteoblast-lineage cells^22,23^. In this study, we found YAP1 expression was downregulated in osteoporosis specimens and upregulated in hBMSCs during osteogenic differentiation. Furthermore, YAP1 was a target of miR-214, and the inhibitory effects of miR-214 on hBMSCs osteogenic differentiation could be reversed by YAP1 overexpression. This is the first study to demonstrate that miR-214 suppresses the osteogenic differentiation of hBMSCs by downregulating YAP1 expression.

In conclusion, the current findings provided evidences that circ-ITCH promoted osteogenic differentiation and alleviated symptoms of osteoporosis in the OVX mice model by sponging miR-214 to upregulate YAP1 expression. Our results revealed a new mechanism of osteogenic differentiation, and further provided a novel therapeutic strategy for treating osteoporosis. However, more research is required to clarify the upstream regulatory mechanism of how circ-ITCH downregulated in osteoporosis. We will also keep working to prove whether circ-ITCH regulates other bone degeneration diseases, such as osteoarthritis and bone senescence.
